# Editorial: Trustworthy AI for healthcare

**DOI:** 10.3389/fdgth.2024.1427233

**Published:** 2024-06-03

**Authors:** Oleg Agafonov, Aleksandar Babic, Sonia Sousa, Sharmini Alagaratnam

**Affiliations:** ^1^ Healthcare Programme, Group Research and Development, DNV, Høvik, Norway; ^2^ School of Digital Technologies, Tallinn University, Tallinn, Estonia

**Keywords:** systems, patient monitoring, robotics, personalized medicine, drug discovery, clinical trials, monitoring

**Editorial on the Research Topic** Trustworthy AI for healthcare

Artificial Intelligence (AI) integration in healthcare has been met with enthusiasm but also caution (1). The expectation of AI to revolutionize healthcare is high, with its potential to enhance access, improve quality, and streamline efficiency. The landscape of AI in healthcare is rapidly evolving, with significant advancements in diagnostics, decision support systems, patient monitoring, robotics, personalized medicine, drug discovery, clinical trials, monitoring, and organizational workflow management (2). However, the adoption of AI in healthcare has not kept pace with its development. This is often attributed to the lack of various aspects of trustworthiness in AI systems (3–5).

This research topic explores the concept of “trustworthy AI for healthcare,” which stands at the intersection of technology, ethics, and clinical practice. Trustworthy AI for healthcare refers to the development and deployment of AI systems in healthcare that are reliable, safe, and transparent, and that respect ethical principles and values. It delves into the current state of AI in healthcare, the challenges impeding its adoption, and the paramount importance of trust. It underscores the need for transparency in AI algorithms, the ability to interpret and explain AI decisions, and the collaborative efforts required to achieve these goals (6, 7).

The article “Dicing with data: the risks, benefits, tensions and tech of health data in the iToBoS project” reviews the iToBoS project, which created an AI tool for early melanoma detection. Key challenges identified in the project include (1) a small clinical trial cohort, raising anonymization concerns; (2) difficulty in obtaining informed consent due to the necessity to explain the involved complex technology; (3) an open data commitment, necessitating extra privacy measures for diverse data types; and (4) communicating algorithmic results to stakeholders. The authors reflect on the tensions that these issues cause, considering the broader health sector challenges.

The authors of the article “Developing machine learning systems worthy of trust for infection science: A requirement for future implementation into clinical practice” discuss the critical role of infection science, particularly during the SARS-CoV-2 pandemic, and emphasize the potential of AI in improving patient outcomes, optimizing clinical workflows, and enhancing public health management. Despite promising research, the lack of trustworthy AI systems hinders the transition of AI models from research to clinical practice. The paper advocates for developing systems that meet user, stakeholder, and regulatory requirements and highlights the need for a systematic approach to trustworthiness in AI applications.

In the article “The unmet promise of trustworthy AI in healthcare: why we fail at clinical translation,” the authors state that the clinical application of AI often fails, primarily due to the lack of a precise definition of “trust” and “trustworthiness.” This deficiency leads to unintentional misuse and the possibility of intentional “thics washing” by industry stakeholders. The paper contends that these barriers hinder the realization of trustworthy medical AI’s potential and advocates for reassessing the meaning of trust in healthcare AI to close the gap between theoretical guidelines and practical application.

The authors of the article “A trustworthy AI reality-check: the lack of transparency of artificial intelligence products in healthcare” state that trustworthiness hinges on transparent algorithm development and testing to pinpoint biases and communicate harm risks. Publicly available information on the risks of medical AI products is often insufficient. This study assessed 14 CE-certified AI radiology products in the EU, examining their transparency based on a custom survey aligned with AI trust guidelines. The transparency scores varied widely; and significant gaps in training data documentation, ethical considerations, and deployment limitations were found. The authors call for establishing transparency requirements to uphold AI’s trustworthiness in healthcare.

Trust among various stakeholders—societies, organizations, and businesses—is crucial for ensuring smooth operations, creating value, and minimizing disruptions or accidents. However, existing regulations often do not fully cater to technological advancements, leading to gaps that may introduce challenges. In response, newly introduced regulations, such as the European Union’s Artificial Intelligence Act (AI Act) and the U.S. Executive Order on Artificial Intelligence, aim to set requirements for trustworthy and responsible AI. The development of trustworthy AI is imperative to foster a healthcare environment where AI can be safely, scalably, and sustainably adopted.

Standards play a crucial role in bridging the gap between the high-level principles outlined in regulations that cover all sectors and the concrete technical specifications needed for AI-enabled systems to achieve compliance in specific sectors. To build trust in these systems, regulations and legislation may require third-party audits to provide assurance that entities comply with the established standards. As regulations and standards evolve, the role of ongoing research also becomes increasingly critical. Research on Trustworthy AI is essential not only for shaping these emerging regulations but also for providing the vital insights and empirical data needed to inform and refine both regulations and the corresponding standards.

Collaboration between academia and industry is crucial in this endeavor. Each brings to the table a wealth of knowledge and experience that is both distinct and complementary. Industry provides practical insights from deploying and implementing AI systems, while academia contributes through rigorous research and theoretical frameworks. This synergy can accelerate the adoption of AI systems that are not only innovative but also reliable and understandable.

In conclusion, integrating AI into healthcare is complex and fraught with challenges. Yet, the pursuit of trustworthy AI is a journey worth undertaking. It promises a future where healthcare is not only powered by intelligence but also grounded in trust—a future where AI acts as a partner in healthcare delivery, augmenting clinicians’ capabilities and enhancing patient care. This editorial calls for a multidisciplinary approach to realize this vision, urging stakeholders across industry and academia to unite in the quest for an AI-enabled healthcare system that is as trustworthy as it is transformative.
